# Summer Complaint

**Published:** 1879-11

**Authors:** 


### SUMMER COMPLAINT.

This means a diarrhœa, a looseness of the bowels of infants and young children, which prevails to such an extent in midsummer, especially in large cities, that the number of deaths is doubled. This is not necessarily the result of warm weather; in reality there ought to be fewer deaths among children in warm weather than in cold, because they can be more out of doors. The universal cause of "summer complaint" is injudicious feeding, and this not so much as to quality or quantity as in frequency. Children are not apt to over-eat, no more so than the brute creation, if they are fed at regular times. If mothers will only take pains to feed their little ones at regular times and at proper intervals—if this were done for one single summer—there would be thousands of little ones saved.

If a child is fed—if it is allowed to eat as much as it wants—a certain time is required for this food to be digested, dissolved, and passed out of the stomach, and then it begins to get hungry again, the hunger gradually increasing until it becomes painful if not satisfied. In grown persons it requires five hours for a regular meal to be fully digested, hence the regular meals of a family should not be less than five hours apart, during which interval not an atom of food should be eaten, ordinarily, because it has been found by ocular observation that if, after a regular meal (an hour or more) something else is eaten, the process of digestion of what was previously eaten is arrested until what is eaten later has been put into the condition of what was eaten before—precisely as when a lump of ice is put into a vessel of boiling water it ceases to boil until the ice has been brought to the boiling point.

